# Refractory and Relapsing Laryngeal Edema Possibly Associated With Chronic Tonsillitis and Mycoplasma Infection, Requiring Reintubation and Tracheostomy

**DOI:** 10.1155/carm/6638796

**Published:** 2025-01-04

**Authors:** Yutaka Tsukamoto, Takashi Sugimoto, Masataka Umeda, Yuki Furuse, Haruo Yoshida, Yuka Nagae, Yasuo Ohsato, Yukitaka Ueki, Maeda Takahiro, Koya Ariyoshi

**Affiliations:** ^1^Department of Clinical Medicine, Institute of Tropical Medicine, Nagasaki University, Nagasaki, Japan; ^2^Department of Immunology and Rheumatology, Nagasaki University Graduate School of Biomedical Sciences, Nagasaki, Japan; ^3^Department of General Medicine, Nagasaki University Graduate School of Biomedical Sciences, Nagasaki, Japan; ^4^Medical Education Development Center, Nagasaki University Hospital, Nagasaki, Japan; ^5^Department of Otolaryngology-Head and Neck Surgery, NHO Nagasaki Medical Center, Nagasaki, Japan; ^6^Department of Respiratory Medicine, Sasebo Chuo Hospital, Sasebo, Nagasaki, Japan; ^7^Department of Otolaryngology, Sasebo Chuo Hospital, Sasebo, Nagasaki, Japan; ^8^Rheumatic and Collagen Disease Center, Sasebo Chuo Hospital, Sasebo, Nagasaki, Japan

**Keywords:** chronic tonsillitis, laryngeal edema, Mycoplasma, relapsing epiglottitis

## Abstract

Relapsing epiglottitis has rarely been reported, and its etiology is not well established. A 44-year-old previously healthy Japanese man presented with a quickly progressing choking sensation. He had been experiencing refractory and relapsing laryngeal edema and probably acute epiglottitis (three episodes within 2 weeks), with rash and elevated pancreatic amylase. The patient required immediate intubation. After the initial extubation, he required reintubation and a subsequent tracheostomy. Antibiotics, glucocorticoid, and antihistamines were administered, and he finally recovered with the tracheostomy's closure. Potential causes of this patient's relapsing epiglottitis are as follows: persistent right swollen tonsil; a positive result on a Mycoplasma pneumoniae antigen test and a particle agglutination (PA) test, implicating chronic tonsillitis; and/or Mycoplasma infection. This is the first case report of refractory and relapsing epiglottitis requiring reintubation possibly concurrent with chronic tonsillitis and Mycoplasma infection.

## 1. Introduction

Acute epiglottitis is a potentially life-threatening disease which can lead to upper airway obstruction. A study conducted in Denmark described an incidence at 0.02 cases/100,000 per year in children and 1.9 cases/100,000 per year in adults [1]. Only a few case reports of recurrent epiglottitis have been published [2–7]. Hemophilus influenzae type B (Hib) had shown to be a major causative agent of acute epiglottitis [8], and the incidence of acute epiglottitis decreased dramatically after the introduction of the conjugate Hib vaccine. Other causative organisms include *Streptococci*, *Staphylococcus aureus*, *Neisseria meningitidis*, and *Prevotella* species [8, 9]. However, to the best of our knowledge, there are no published case reports of acute epiglottitis caused by Mycoplasma pneumoniae.

We encountered a patient with refractory and relapsing laryngeal edema that was probably epiglottitis. He had experienced multiple relapses within a 2-week period, whereas the previously reported patients with recurrent epiglottitis had experienced each episode over periods of months to years, and the patients' respective symptoms developed after the complete resolution of a past illness [2–7]. Our patient's case indicated the existence of chronic tonsillitis and Mycoplasma infection as possible underlying causes.

## 2. Case Presentation

A 44-year-old previously healthy Japanese man was transferred to a hospital by emergency medical service during the daytime, complaining of a 2-h course of rapidly deteriorating difficulty breathing. He had experienced choking sensations during his work as a plumber at a new worksite, but he denied throat pain, diarrhea, and accidental ingestions. He reported that he had experienced a rash and itchiness in cold temperatures that had begun 2 years prior to this transfer. He noted that his child had experienced a persistent mild cough 1 week earlier. He had no known allergies and was not using any medications. His vaccination history was not clear, but most people in his age in Japan are immunized for diphtheria.

**Figure 1:**
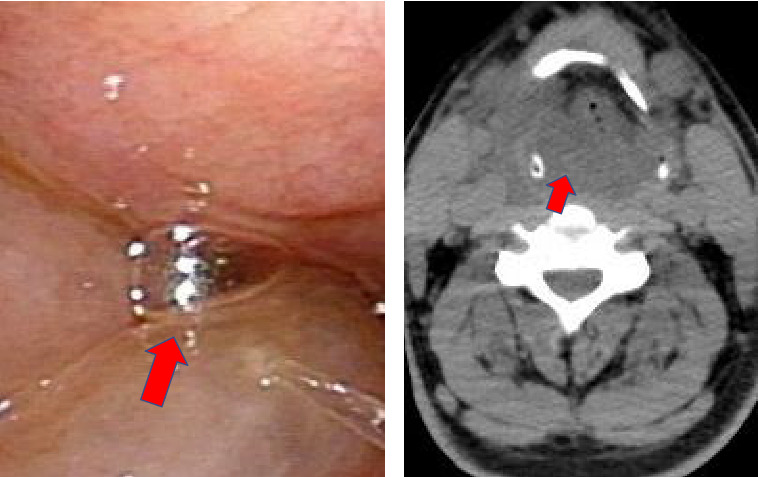
Laryngoscopy (a) and computed tomography (b) on the admission of the patient, a previously healthy 44-year-old male. His larynx was surprisingly swollen (red arrows).

His height and weight were 169 cm and 68 kg, respectively. On arrival at the hospital, his vital signs were remarkable for a respiratory rate at 30 breaths per minute, percutaneous oxygen saturation (SpO_2_) 100% on 10 L per minute supplemental oxygen with a mask, blood pressure at 178/114 mmHg, and body temperature at 39.0°C. He had severe difficulty breathing, but his Glasgow Coma Scale result was E4V5M6. We observed marked stridor, and he could not speak or lie down flat. We did not detect any pseudomembrane in his mouth. His breathing difficulty was rapidly worsening, and we anticipated impending suffocation. We thus immediately intubated him with bronchoscopy, which revealed severe pharyngeal and laryngeal edema (Figure 1). He was then admitted to the hospital's intensive care unit. He had no rash, generalized edema, or abdominal pain at that time.

The initial laboratory investigation was notable for leukocytosis (white blood cell count 10.5 × 10^9^/L, neutrophils 9.3 × 10^9^/L, lymphocytes 0.84 × 10^9^/L, eosinophils 0.021 × 10^9^/L). C-reactive protein (CRP) was slightly elevated at 0.17 mg/dL, and the results of a liver function test and kidney function test were normal. A severe acute respiratory syndrome coronavirus 2 antigen test and polymerase chain reaction (PCR) test were negative. The patient's results on a Mycoplasma pneumoniae rapid antigen test (Ribotest Mycoplasma, Asahi Kasei Pharma, Tokyo) was positive, and his particle agglutination (PA) test (Serodia Myco II, Fujirebio, Tokyo) result was significantly elevated at 1:640 (considered clinically significant when ≥ 1:160) on admission (1:40 on Day 11); however, the result of a Mycoplasma pneumoniae PCR test for tracheal tube aspirate was negative.

Two sets of blood culture were negative, and the culture of the patient's tracheal tube aspirate, which was not purulent, showed normal oral flora. Computed tomography (CT) revealed severe swelling of the pharynx and larynx (right-side dominant), but there was no evidence of a tumor, abscess, or pneumonia. The patient's airway was almost completely obstructed (Figure 1).

**Figure 2:**
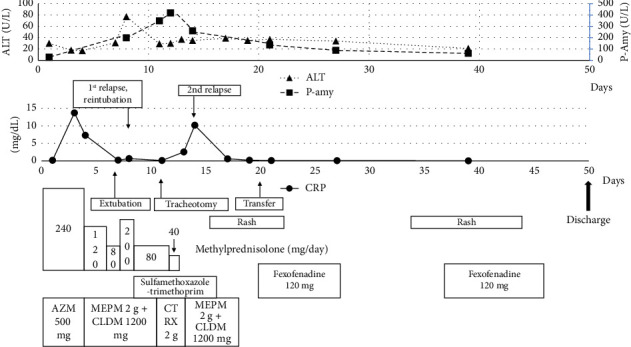
The patient's clinical course during admission. ALT: alanine transaminase; AZM: azithromycin; CLDM: clindamycin; CRP: C-reactive protein; CTRX: ceftriaxone; MEPM: meropenem; P-Amy: pancreatic amylase.

After the patient's admission, we administered methylprednisolone, antibiotics, and bronchodilators (salbutamol and adrenaline), as shown in Figure 2. The pharynx and larynx swelling was then relieved, and the patient was extubated on Day 7. However, the swelling worsened again on Day 8, and he was reintubated. He complained of throat pain. We also detected elevated pancreatic amylase without abdominal pain at this point, and this value was highest on Day 13 at 451 U/L. We observed no sign of pancreatitis on abdominal CT. The patient's throat inflammation was improved on Day 9, but we performed a tracheotomy on Day 11 because he was experiencing repeated airway obstruction. We evaluated his swallowing function with water on Day 12; it was normal.

**Figure 3:**
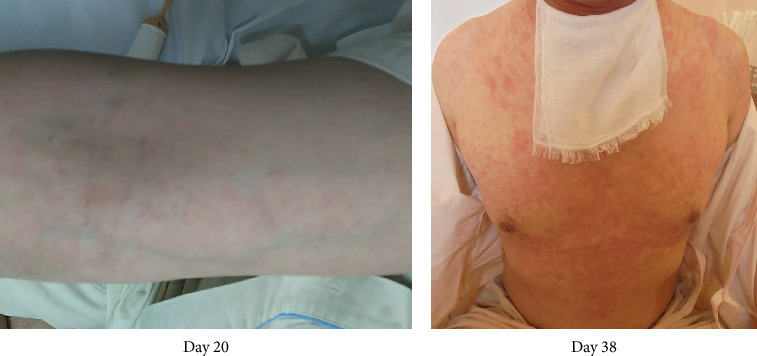
The patient's skin rash on Days 20 (a) and 38 (b).

**Figure 4:**
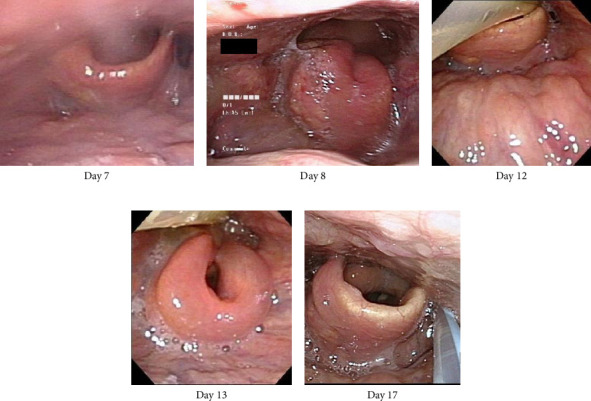
The appearance of the changes in the patient's epiglottis.

**Figure 5:**
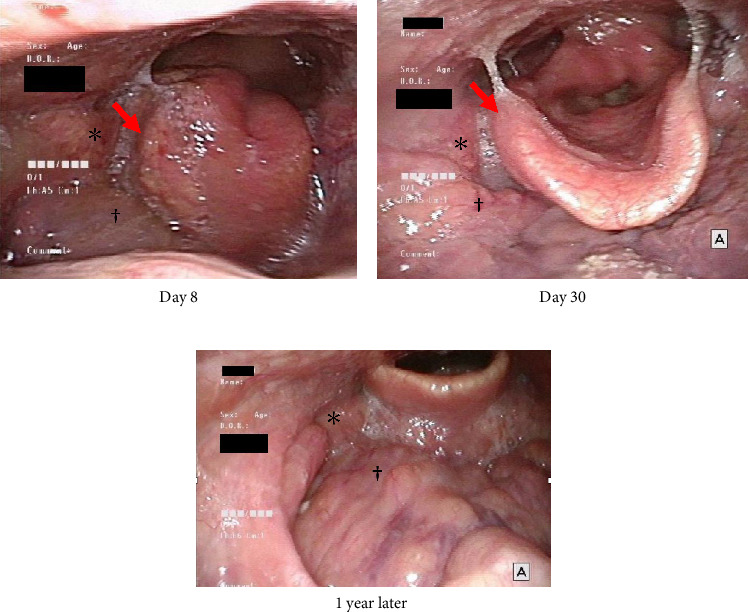
Endoscopic findings of the patient's right palatine (⁣^∗^) and lingual (^†^) tonsillitis. (a) Acute tonsillitis and swelling and erosion of the right side of the epiglottis were observed (arrow). (b) The swelling of the right side of the epiglottis improved almost completely, and the erosion disappeared. The acute tonsillitis improved but mildly persisted. (c) Mild edematous swelling of the epiglottis persisted, but the right lingual and palatine tonsillitis completely resolved (distant view).

On Day 13, the patient exhibited a fever, leukocytosis, and epiglottis swelling again, and these resolved 2 days later. By Day 18, he had gradually developed generalized erythema (Figure 3), and we discontinued all of the medications and prescribed fexofenadine and topical glucocorticoid for possible allergic etiology. As the etiology was not clear, the patient was transferred to a university hospital for a comprehensive investigation on Day 20. A biopsy of the anterior wall of the right oropharynx was performed on Day 25, and the histological results showed low-grade/mild dysplasia but no malignancy. The rash disappeared within a week but appeared again on Day 34. This rash did not respond to fexofenadine or steroid ointment, but it gradually improved over approx. 10 days. The patient showed no subsequent laryngeal swelling since that time point, and the tracheostomy was closed on Day 41. He was discharged on Day 49 and had no relapse for 12 months. The mild swelling of the right lingual tonsil persisted but gradually resolved. The palatine tonsil swelling was not observed. Figures 4 and 5 depict the changes in the patient's epiglottis and tonsil swelling.

We investigated the causes of relapsing laryngeal edema. Angioedema and allergic reaction were differential diagnoses in addition to epiglottitis. Regarding angioedema, the patient's C4 value was 23.8 mg/dL (reference range [ref.] 11–31 mg/dL), and the C1 inhibitor level was 147% (ref. 70%–130%). These results made hereditary or acquired angioedema less likely. The patient's nonspecific immunoglobulin (Ig) *E* value was 173 U/mL (ref. < 250), and radioallergosorbent tests (RASTs) were positive for both Dermatophagoides farina (house dust mites) at 19.3 U/mL, Class IV and house dust at 16.1 U/mL, Class IV. The results of drug-induced lymphocyte stimulation tests for meropenem and clindamycin were negative. Immunological test results were unremarkable: IgA 259 mg/dL, IgG 924 mg/dL, IgM 112.7 mg/dL, and no monoclonal protein. The patient's spleen was normal, and the human immunodeficiency virus screening test result was negative. A blood test for Epstein–Barr virus (EBV) was done on Day 14, and the test result for antiviral capsid antigen (VCA) IgM was negative, and those for anti-VCA IgG and EBV nuclear antigen (EBNA) were positive.

## 3. Discussion

Several case reports of recurrent acute epiglottitis are available [2–7], but our literature search identified no articles about relapsing epiglottitis occurring within a short period. The reported patients had recurrent episodes over periods of months to years, whereas our patient had two relapses within 2 weeks, resulting in reintubation and tracheostomy. Our literature search did not identify any cases of patients with epiglottitis who required reintubation. Even if a patient's laryngeal swelling improves, he or she should be closely monitored for the possibility of relapse.

This case apparently provides the first published report of refractory and relapsing epiglottitis that occurred within a short period. The remarkable swelling of his larynx, the throat pain, and the elevated CRP suggested an infectious disease and supported the diagnosis of acute epiglottitis. We discontinued methylprednisolone on Day 12 of his hospitalization (with a gradual taper); he experienced the first relapse with high-dose glucocorticoid, and his symptoms resolved after the second relapse without steroid readministration. Considering this, we speculate that the steroid treatment did not have a significant influence on the patient's clinical course.

Regarding the underlying causes of his acute epiglottitis, chronic tonsillitis and Mycoplasma infection might be possible. This patient had throat swelling with right-side dominance, and the mild swelling still persisted after symptom resolution. One report described a 20-year-old woman who experienced acute epiglottitis three times over a 3-year period with persistently hypertrophic lingual tonsils and no recurrence after tonsillectomy [7]. We suspect that our patient (i) had underlying continuous right tonsillitis and (ii) some precipitating factors made the condition worse.

Mycoplasma pneumoniae has never been described as a causative agent of acute epiglottitis [8], but in our patient's case, the interpretation of the Mycoplasma pneumoniae test results was unclear. Hib and *Streptococci* are the major causes of acute epiglottitis, but our patient's tracheal tube aspirate culture showed normal flora. His result on a Mycoplasma pneumoniae rapid antigen test was positive, and his PA test level was remarkably elevated at 1:640 and clinically significant. However, it was 1:40 on Day 11, and the tracheal tube aspirate culture PCR test result for Mycoplasma pneumoniae at that time was negative. Although the PA test and rapid antigen test have relatively high specificity at 90% [10–13], the diagnosis was not definite.

There are several possible reasons for this. (1) It is not known which type of specimen (sputum, tracheal tube aspirate, pharyngeal swab, and epiglottic swab) is the best for the diagnosis of pathogens for acute epiglottitis. We should have obtained specimens from the patient's pharynx and/or larynx. (2) It is not clear whether high-dose glucocorticoid can affect the PA test. (3) As described [14], infection with another Mycoplasma species, not Mycoplasma pneumoniae, and cross-reactivity might have happened. (4) Inconsistent results between PCR and serology test results have been reported [15], which suggests that there may be no reliable tests for diagnosing Mycoplasma pneumoniae infection.

No clear causes of our patient's skin rash and elevated pancreatic amylase were identified, and Mycoplasma pneumoniae infection can be a cause of both [16]. The skin and mucous lesion in his case may have partly overlapped with Mycoplasma-induced rash and mucositis (MIRM), but the skin lesion and severe laryngeal swelling was not typical. MIRM is a novel entity characterized by skin eruption and mucositis associated with Mycoplasma pneumoniae infection, and it sometimes recurs [17]. Suggested mechanisms of MIRM include polyclonal B-cell proliferation and antibody production, and the molecular similarity between Mycoplasma pneumoniae P1-adhesion molecules and a keratinocyte antigen [18]. These allergic reactions could explain the rapid worsening in our patient's case. Since Mycoplasma pneumoniae causes upper respiratory infection [19], theoretically it can evoke infection around the larynx (including epiglottitis). His fever, elevations of the white blood cell count and CRP as well as the poor response of the skin rash to antihistamines and glucocorticoid ointment suggested an infection rather than an allergy or immunological disorder. We are thus unable to conclude whether Mycoplasma infection played a role in his case, but it is possible that its infection was a coexisting condition. The reported prevalence of macrolide-resistant Mycoplasma pneumoniae in Japan is 43.6%–81.6% [20], and this resistance might have affected our patient's poor clinical response to the 3-day course of azithromycin therapy.

Other differential diagnoses may include angioedema and anaphylaxis. Normal values of C4 and C1 inhibitors are not suggestive of hereditary or acquired angioedema. The present patient was not using any medication, including angiotensin-converting enzyme inhibitors and nonsteroidal anti-inflammatory drugs, which can be related to angioedema. We consider anaphylaxis less likely in his case because he did not have skin lesions, hypotension, or gastrointestinal symptoms at the first presentation, and he denied any exposure to possible allergens including foods, drugs, and dust. One report suggested an association between recurrent epiglottitis and immunological abnormalities [4], but we did not detect such disorders in our patient.

We have described what appears to be the first case report of a patient with refractory and relapsing epiglottitis that occurred within a short period of time who needed reintubation and tracheostomy. Refractory and relapsing epiglottitis cases are rare, and the etiology is not well established. We could not make a definitive conclusion in the present patient's case, but chronic tonsillitis and Mycoplasma infection might have been coexisting factors affecting the clinical course. The potential involvement of Mycoplasma infection in patients with acute epiglottitis is clinically important, as third-generation cephalosporins are generally used as a first-line treatment and they are not effective for Mycoplasma species. This case report has a limitation in terms of diagnostic tests for Mycoplasma infection. Future prospective studies are necessary to clarify the mechanism underlying refractory throat swelling, and the findings of such studies could lead to better management and understanding of refractory and relapsing epiglottitis.

## 4. Conclusion

We have described a case of refractory and relapsing epiglottitis involving three episodes within a 2-week period, possibly concurrent with chronic tonsillitis and Mycoplasma infection, in a patient who required reintubation and tracheostomy. Future case series and prospective studies are necessary to clarify the etiology of this order, which could lead to the discovery of novel management strategies for refractory laryngeal edema.

## Data Availability

The data that support the findings of this study are available from the corresponding author upon reasonable request.
